# Targeting aggressive prostate cancer-associated CD44v6 using phage display selected peptides

**DOI:** 10.18632/oncotarget.21421

**Published:** 2017-09-30

**Authors:** Ying Peng, Austin R. Prater, Susan L. Deutscher

**Affiliations:** ^1^ Research Service, Harry S. Truman Memorial Veterans Hospital, Columbia, MO, USA; ^2^ Department of Biochemistry, University of Missouri-Columbia, Columbia, MO, USA

**Keywords:** prostate cancer, CD44v6, peptide, phage display, biomarker

## Abstract

There is a crucial need to identify new biomarkers associated with aggressive prostate cancer (PCa) including those associated with cancer stem cells (CSCs). CD44v6, generated by alternative splicing of CD44, has been proposed as a CSC biomarker due to its correlation with aggressive PCa disease. We hypothesized that phage display selected peptides that target CD44v6 may serve as theranostic agents for aggressive PCa. Here, a 15 amino acid peptide (“PFT”) was identified by affinity selection against a peptide derived from the v6 region of CD44v6. Synthesized PFT exhibited specific binding to CD44v6 with an equilibrium dissociation constant (Kd) of 743.4 nM. PFT also bound CD44v6 highly expressed on human PCa cell lines. Further, an aggressive form of PCa cells (v6A3) was isolated and tagged by a novel CSC reporter vector. The v6A3 cells had a CSC-like phenotype including enriched CD44v6 expression, enhanced clonogenicity, resistance to chemotherapeutics, and generation of heterogeneous offspring. PFT exhibited preferential binding to v6A3 cells compared to parental cells. Immunohistofluorescence studies with human PCa tissue microarrays (TMA) indicated that PFT was highly accurate in detecting CD44v6-positive aggressive PCa cells, and staining positivity was significantly higher in late stage, metastatic and higher-grade samples. Taken together, this study provides for the first time phage display selected peptides that target CD44v6 overexpressed on PCa cells. Peptide PFT may be explored as an aid in the diagnosis and therapy of advanced PCa disease.

## INTRODUCTION

PCa remains the second leading cause of cancer death for men in the United States, with an estimated 161,360 new diagnosed cases and 26,730 deaths in 2017 [1]. PCa patients with localized disease have favorable long-term survival outcomes due to advances in surgical resection, androgen deprivation therapy (ADT) and chemotherapy [2]. However, patients with aggressive tumors have a poor prognosis and up to 30% of these patients suffer a relapse within 18 months after surgical resection and ultimately die from disease [3]. The effectiveness of chemotherapeutics is challenged by drug resistant subpopulations within the prostate tumor [4, 5]. While initially responsive to ADT, almost all PCa patients will inevitably progress to recurrent castration-resistant prostate cancer (CRPC) and die from more aggressive secondary diseases [6, 7]. Thus, there is a crucial need to identify biomarkers associated with aggressive PCa and to develop new methods to target these tumors.

The hyaluronic acid (HA) receptor CD44 is a highly glycosylated protein that is crucial in cell adhesion, proliferation, differentiation and migration of healthy cells [8, 9]. The CD44 genomic loci, encoding a 742 amino acid protein, consists of 20 exons of which the first and last five exons are conserved (CD44), whereas the 10 exons located between these regions are subject to alternative splicing, resulting in the generation of variable extracellular domains within the CD44 molecule (CD44v1-10) [10]. Most cells express CD44 that requires activation before binding to HA, ensuring cell anchorage to the extracellular matrix. However, under pathological conditions alternative splicing results in expression of continuously active high molecular weight structurally heterogeneous isoforms (CD44v1-10) that are overexpressed in certain cancers [8, 9]. CD44v6, which is generated by the addition of 45 amino acids in variant domain 6 (v6) to the conserved domain of CD44, has been studied as a potential cancer biomarker due to its positive correlation with increased tumor progression as well as metastatic potential of several human cancers including pancreatic, breast, colorectal, ovarian and gastric cancers [11–15].

Results of recent studies have shown high levels of CD44v6 expression in 90% of highly aggressive primary PCa tissues and 100% of lymph node metastases [16]. *In vitro* experiments demonstrated that the knock down of CD44v6 in PC3M, DU145 and LNCap cells suppressed PCa cell proliferative, invasive and adhesive abilities, reduced sphere formation, enhanced chemo-/radiosensitivity, and down-regulated epithelial-mesenchymal transition [16]. In a clinical study, expression levels of CD44, CD44v6, and CD44v10 in radical prostatectomy specimens from 160 patients with clinically localized PCa were evaluated by immunohistochemical staining [17]. High expression of CD44v6, but not that of CD44 or CD44v10, was found to be significantly related to advanced pathological stage and high incidence of seminal vesicle invasion [17]. These findings indicate that CD44v6 is likely involved in the proliferation and progression of aggressive PCa. Therefore, molecular probes specifically targeting CD44v6 may be a promising tool for the challenging task of early detection and targeted treatments of aggressive prostate tumors.

During the past decade, the investigation of the differential expression of human CD44v6 was enabled by the development of murine monoclonal antibody (mAb) specific for epitopes encoded by the variant exon v6 [18]. The majority of these antibodies (Ab) were suitable for the immunohistochemical detection of CD44v6 both in frozen and formalin-fixed, paraffin-embedded tumor tissues [18]. However, clinical application of these mAbs in cancer imaging and therapy has been hampered because of their large size (150 kDa), which results in slow clearance, limited tumor penetration and high liver uptake. For example, a clinical trial of bivatuzumab, a humanized mAb directed against CD44v6, showed some clinical success; however, the development of this drug was abruptly ended due to skin-related toxicities and even death [19]. As an alternative, chemically synthetic peptides have certain advantages over Abs, such as lower synthetic manufacturing costs, greater stability, minimal immunogenicity and well-established bioconjugation strategies [20, 21]. Peptides have also proved particularly useful for the detection of early tumor lesions because they can be more easily delivered to the location of the carcinoma and can penetrate into the lesion with rapid binding and more predictable pharmacokinetics [22]. Peptide-mediated tumor targeted delivery of conventional chemotherapeutic drugs would promise effective and cost-saving control of disease while attenuating undesirable side effects [20, 21]. Furthermore, tumor targeted peptides can be used as probes for molecular or radio- imaging by delivering contrast molecules or radionuclides [23, 24].

Since its development in 1985 by George P. Smith, bacteriophage (phage) display has become a technology of choice for selecting peptides with specific binding properties [25]. A phage library often displays up to 10^9^ unique peptides and can be screened by *in vitro*, *in situ*, and *in vivo* affinity selection against various targets, including proteins, cells, and organs to identify peptides with the desired properties [26]. Numerous studies have been performed to screen and validate peptide ligands that target cancer cells or the tumor vasculature [27, 28]. However, CD44v6-avid peptides have not been reported. Our laboratory has been particularly successful in using phage display to develop high affinity targeting peptides against a number of cancer associated antigens for clinical assay development as well as optical and radio- imaging and therapeutic purposes [29–35]. We hypothesize that phage display selected peptides that target CD44v6 may serve as theranostic agents for aggressive PCa.

In the present study, phage libraries displaying linear peptides and disulfide-constrained peptides were subjected to affinity selection against a peptide derived from the v6 region of CD44v6 [36]. Numerous phage and corresponding synthetic peptides were analyzed. One selected peptide known as “PFT” specifically bound to CD44v6 in the v6-specific region and did not bind CD44. Further, PFT bound to CD44v6 highly expressed on human PCa cell lines PC3M and MDA-PCa-2b, but did not bind to less aggressive PC3 cells. By utilizing a novel reporter system, we also isolated a more aggressive cell subpopulation from PC3M cells known as v6A3 cells, to determine whether CD44v6 expression and PFT binding correlate with more aggressive versus less aggressive cancer cell subpopulations. We found higher CD44v6 expression as well as PFT binding in v6A3 cells than in the less aggressive parental cells, PC3M. Moreover, our results showed that in patient tissue, PFT effectively bound to CD44v6 highly expressed PCa cells and there was a significant correlation between PFT positivity and aggressive PCa staging and grading. These results indicate that peptide PFT could be used not only for targeting PCa-associated CD44v6 but also for distinguishing aggressive from less aggressive PCa tumors. Further, PFT may be developed into an efficacious probe for targeting CD44v6 *in vitro* as well as *in vivo*.

## RESULTS

### Identification of peptides that bind the v6 region of CD44v6 via affinity selection

**Figure 1:**
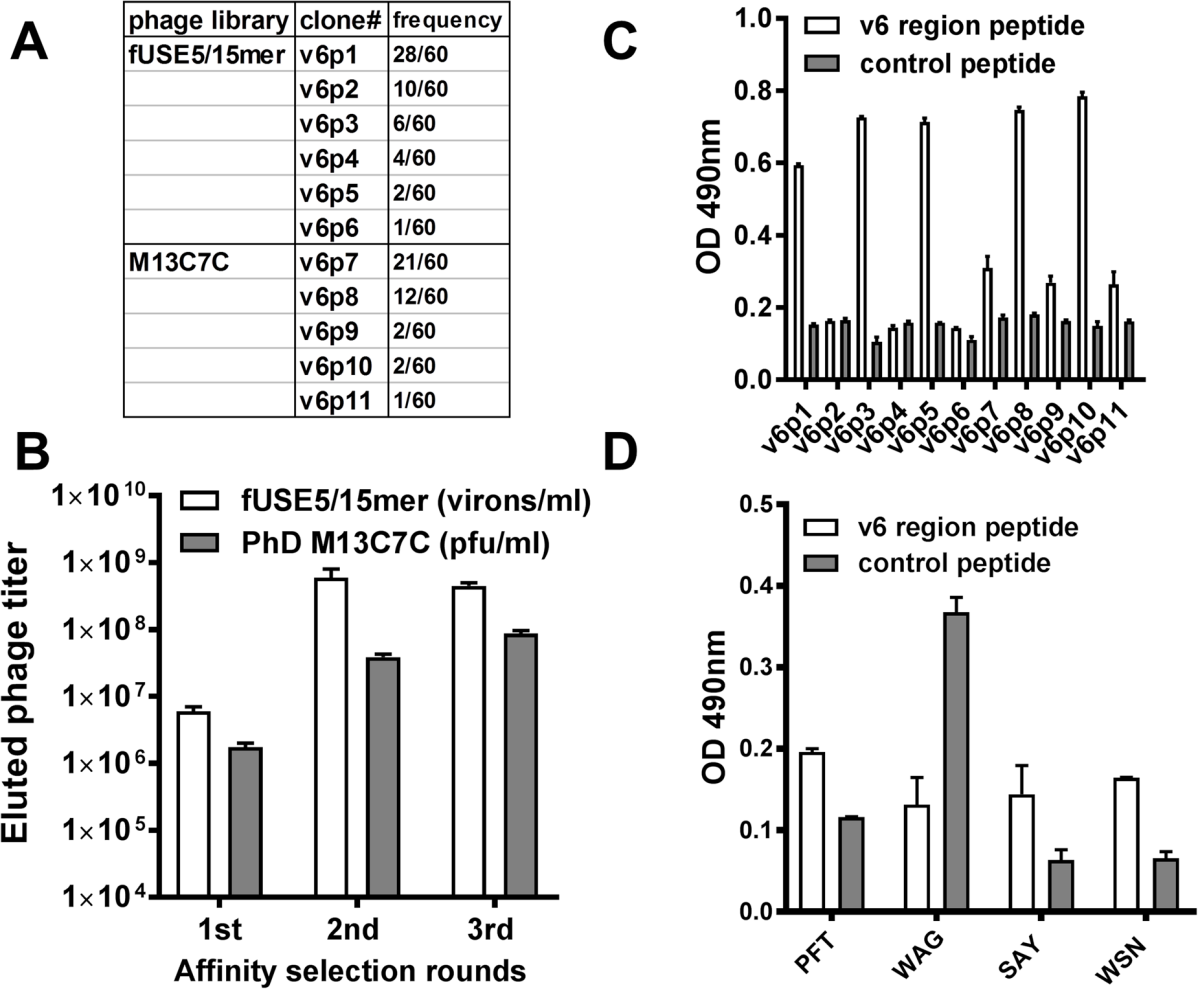
Isolation of CD44v6 avid phage by affinity selection **(A)** The eluted phage titers of each round of affinity selection. The titers of fUSE5/15mer phage (white bar) or M13C7C phage (gray bar) were determined by measurement of virions per milliliter or plaque forming units per milliliter. **(B)** The selected frequency of eleven phage clones with unique sequences. These phage were chosen to test their binding to the v6 region peptide. **(C)** ELISA of the selected phage. After incubation with the immobilized v6 region peptide (white bar) or control peptide (gray bar), the bound phage were detected by a polyclonal HRP-conjugated anti-phage Ab. **(D)** ELISA of synthesized biotinylated peptides. After incubation with the immobilized v6 region peptide (white bar) or control peptide (gray bar), the bound peptides were detected by HRP-conjugated streptavidin.

Potential CD44v6-specific peptides were selected by screening both a random linear 15 amino acid and a cysteine-constrained 7 amino acid peptide library displayed on the N-terminus of coat protein III. Libraries were selected against a v6 region peptide derivative, KEQWFGNRWHEGYR, which encompassed a 14 amino acid sequence of v6 domain of human CD44v6 protein [36]. By the third round of selection, the output phage yield from both libraries was ∼100-fold higher compared with the first round yield (Figure 1A), which indicated enrichment of CD44v6-avid phage clones in the eluted population. After three cycles of affinity selection, sixty individual phage clones from the elution pool of either the fUSE5 or M13C7C library were randomly chosen for DNA sequencing. The obtained DNA sequences were translated into the corresponding peptide amino acid sequences. The PepBank and SAROTUP search databases were used to ensure that the selected peptide sequences were unique to this selection protocol and did not possess known target unrelated peptide sequences (TUPs) [37]. Eleven phage clones with the highest selection frequency and unique sequences were chosen for analysis of their ability to bind the v6 region peptide using phage enzyme linked immunosorbent assay (ELISA) (Figure 1B). As shown in Figure 1C, fUSE5 phage clones v6p1, v6p3, v6p5 and M13C7C phage clones v6p8 and v6p10 exhibited the strongest relative binding to the v6 region peptide. N-terminal biotinylated peptides corresponding to the displayed peptides from phage v6p1 (PFT, PFTVSVPFVWNFTAD), v6p3 (WAG, WAGPRVSSVYYAGAR), v6p8 (SAY, SAYDRPL), and v6p10 (WSN, WSNKGYD), that demonstrated both high selection frequency and superior v6 binding, were synthesized for further *in vitro* characterization (Table 1). Figure 1D indicates PFT, SAY, and WSN peptides exhibited increased binding to v6 region peptide compared to control peptide.

**Table 1:** Peptides chosen for synthesis and biotinylation

Phage clone	Peptide name	Biotinylated peptide sequence
v6p1	PFT	Biotin-GSG-PFTVSVPFVWNFTAD
v6p3	WAG	Biotin-GSG-WAGPRVSSVYYAGAR
v6p8	SAY	Biotin-GSG-SAYDRPL
v6p10	WSN	Biotin-GSG-WSNKGYD

### Characterization of peptide binding to CD44v6

**Figure 2:**
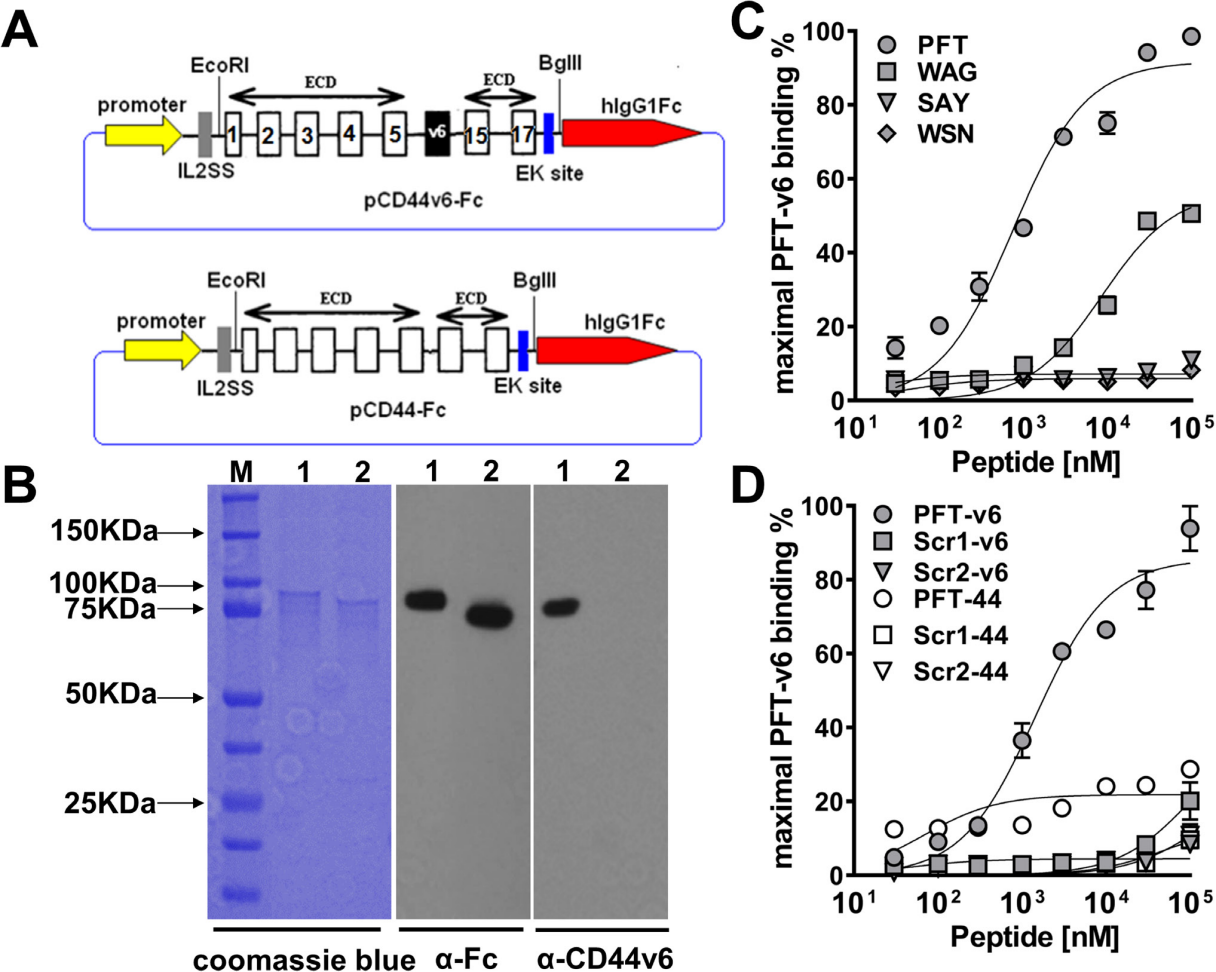
Characterization of peptide binding to recombinant CD44v6 **(A)** Schematic of recombinant CD44v6-Fc and CD44-Fc expression construct. The number of constant exons (1-5 and 15-17) are marked. **(B)** SDS-PAGE analysis and immunoblotting of purified CD44v6-Fc and CD44-Fc (M, BioRad precision plus all blue prestained protein standard; lane 1, CD44v6-Fc; lane 2, CD44-Fc). For SDS-PAGE analysis, the purified proteins were detected by coomassie blue staining. For immunoblotting, the mouse anti-human Fc Ab or mouse anti-human v6 region specific mAb VFF18 was used as the primary Ab and HRP conjugated goat anti-mouse Ab was used as the secondary Ab. **(C)** ELISA analysis of the dose dependent recombinant CD44v6-Fc binding ability of four biotinylated peptides. The peptides displayed on clone v6p1 (PFT), v6p3 (WAG), v6p8 (SAY) and v6p10 (WSN) were synthesized with a biotin label. The maximal ELISA absorbance value of PFT binding to CD44v6-Fc was used to normalize the binding signal of individual peptides. The specific binding curve was generated using GraphPad Prism 6.0 software and absolute Kd values were calculated using nonlinear regression. **(D)** The specificity of PFT binding to recombinant CD44v6-Fc. Different concentrations of PFT and its two scrambled peptide versions were incubated with immobilized CD44v6-Fc or CD44-Fc, respectively for ELISA analyses. The maximal ELISA absorbance value of PFT binding to CD44v6-Fc was used to normalize the binding signal of individual peptides. The specific binding curve was generated using GraphPad Prism 6.0 software.

Because the selection and binding studies thus far were based on phage, it was important to examine whether the displayed peptide could maintain binding once chemically synthesized. To better examine this, recombinant human CD44v6-ECD and CD44-ECD DNA were engineered into pFUSE-hIgG1-Fc2 vector (InvivoGen, San Diego, CA) (Figure 2A) and transfected into CHO-K1 cells. Proteins were produced and purified by protein A affinity chromatography. The identity of the recombinant proteins was confirmed by immunoblotting with VFF18, an anti-human CD44v6-specific mouse mAb and an anti-human fragment crystallizable (Fc) mAb (Figure 2B). As shown, the purified CD44-Fc and CD44v6-Fc proteins yielded a single species by SDS-PAGE analysis and coomassie blue staining with a molecular mass of ∼90 kDa. This size is larger than the predicted molecular weight of 51.5 kDa for CD44-Fc and 57.3 kDa for CD44v6-Fc, which is likely due to glycosylation of the polypeptides (Figure 2B). Binding affinity and specificity of synthetic biotinylated peptides to recombinant CD44v6-Fc was examined by ELISA. Although two fUSE5/15 mer phage clones and two M13C7C phage clones exhibited the strongest relative binding to the v6 region peptide (Figure 1C), our results indicated that only the fUSE5/15mer phage displayed peptides PFT and WAG maintained detectable binding to recombinant CD44v6-Fc once chemically synthesized as linear peptides. As shown in Figure 2C, ELISA analyses demonstrated peptides PFT and WAG bound to recombinant CD44v6 with apparent affinities of 743.4 ±152.7 nM and 8.671 ± 1.854 μM, respectively. Furthermore, no binding of PFT was observed to recombinant CD44-Fc, suggesting binding was occurring in the v6-specific region. In addition, two scrambled sequence versions of PFT, scr1 (VTPVFSFATPNFVDW) and scr2 (VSVWFVTANFPPFTD) were generated by online tools of mimotopes (www.mimotopes.com). Both of these biotinylated scrambled peptides showed negligible binding to CD44v6-Fc (Figure 2D), indicating the binding of PFT was sequence dependent.

### Peptide binding to CD44v6 highly expressed PCa cell lines

**Figure 3:**
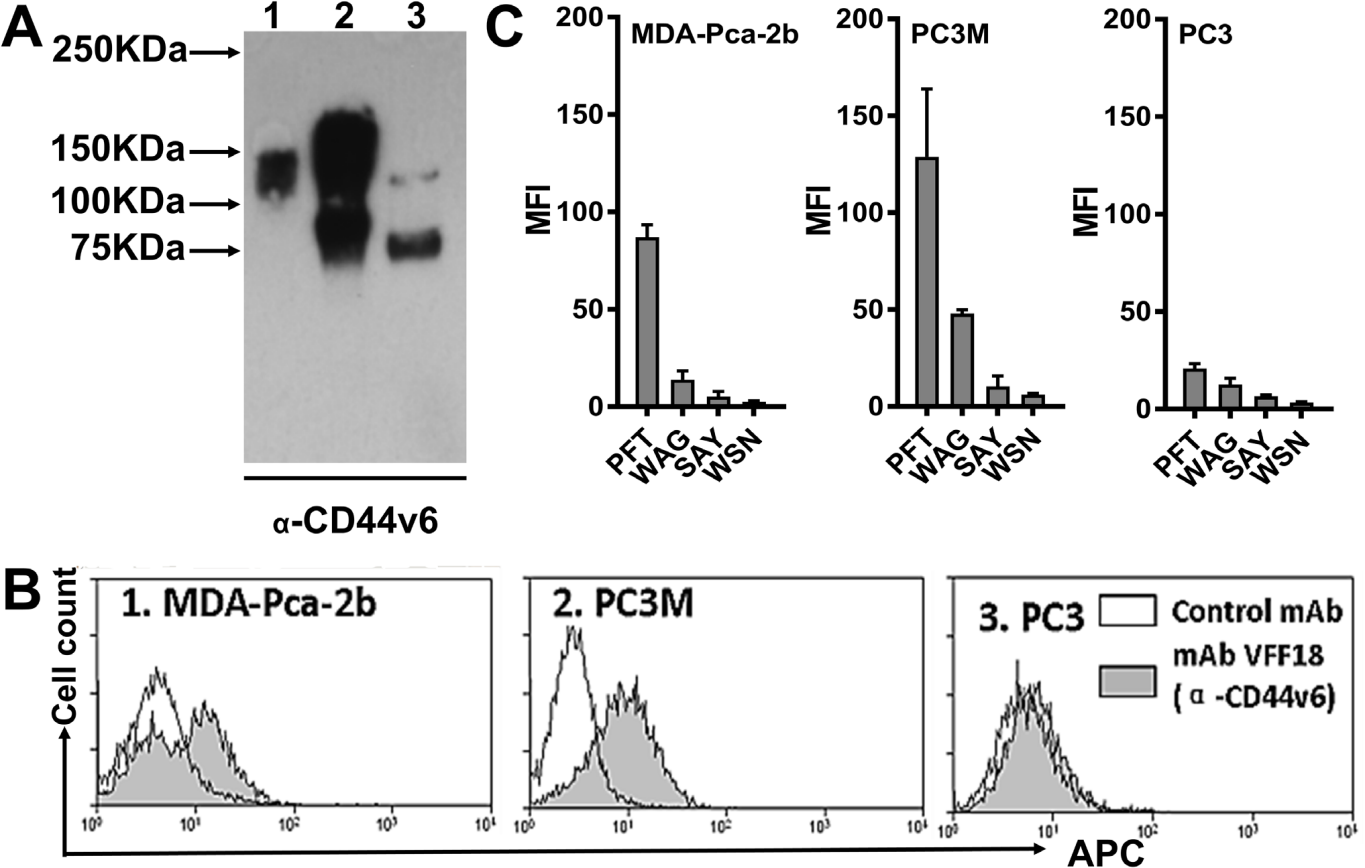
PFT binding to PCa cell lines **(A)** Immunoblotting analysis of CD44v6 expression levels in three human PCa cell lines using the CD44v6-specific mAb VFF18. Lane 1, MDA-PCa-2b; Lane 2, PC3M; Lane 3, PC3. **(B)** A flow cytometry histogram plot of CD44v6 expression on the surface of the three PCa cell lines. The bound VFF18 (gray) or isotype control Ab (blank) were detected by APC conjugated secondary Ab. **(C)** Flow cytometry analysis of the binding of the four peptides (PFT, WAG, SAY and WSN) to the surface of the three PCa cell lines. The bound biotinylated peptides were detected by APC conjugated streptavidin. Bar graphs of quantitative MFI were used to compare the relative cell binding of the different peptides.

The binding of peptide PFT to recombinant CD44v6-Fc suggests that PFT may bind to CD44v6 expressed on the cell surface of human PCa cells. PC3M is a PC3-derived cell line with higher tumorigenic and metastatic properties in nude mice compared to PC3, suggesting PC3M is more aggressive than PC3 cells [38]. It has been well established that PC3M cells do not express the androgen receptor (AR) and prostate specific antigen (PSA) [39]. In contrast, MDA-PCa-2b is an androgen-dependent human PCa cell line derived from a bone metastasis that expresses AR and PSA, representing another aggressive subtype of PCa cells [33]. However, early studies demonstrated that both PC3M and MDA-PCa-2b are CD44v6 overexpressing cell lines [16]. Thus, these cell lines could be used to represent CD44v6 overexpressed in clinically relevant human PCa cells. To ascertain relative CD44v6 expression levels on the cell surfaces of various PCa cell lines, immunoblotting and flow cytometry analysis using the VFF18 mAb specific for CD44v6 were performed. As shown in Figure 3A and 3B, both immunoblotting and flow cytometry analyses demonstrated that the expression of CD44v6 was found to be relatively high in human MDA-PCa-2b and PC3M cells, but moderate in the less aggressive PC3 cell lines. Additionally, different molecular weight CD44v6 species were detected in these cells, consistent with variable glycosylation of CD44v6. Flow cytometry analysis also showed that compared to the other three potential v6-binding peptides (WAG, SAY and WSN), PFT exhibited significantly stronger binding to aggressive cell lines MDA-PCa-2b and PC3M compared to the less aggressive PCa cell line PC3. (Figure 3C), indicating PFT may be valuable in targeting CD44v6 highly expressed PCa cells.

### Transfection of PC3M cells using a NANOG reporter system

As shown in Figure 3B, flow cytometry results indicated that only ∼50% of PC3M or MDA-PCa-2b cells were CD44v6 positive, suggesting some subpopulations of the PCa cells maybe more aggressive than others. Therefore, a major aim in the present study was to isolate and mark the aggressive PCa cells to determine whether CD44v6 expression and PFT binding correlate with more aggressive versus less aggressive cancer cell counterparts. Because CSCs exhibit increased aggressiveness, aggressive cancer cells are most commonly identified and sorted by flow cytometry using combinations of CSC cell-surface markers such as CD44, CD133 and EpCAM [40, 41]. However, optimal marker combinations are not universally applicable because of the heterogeneity within CSC populations. Although the origins of CSCs are debated, accumulating evidence supports the notion that embryonic stem cell (ESC) transcription factors such as NANOG serve as neoplastic engines driving oncogenesis, including those of aggressive PCa [42–46]. Transcription factors essential for CSC stemness, such as NANOG, could be more universal and stable than differentiated and/or redifferentiated surface biomarkers. Therefore, the transcriptional activity of the NANOG promoter could be used for tracking and isolating aggressive PCa subsets.

**Figure 4:**
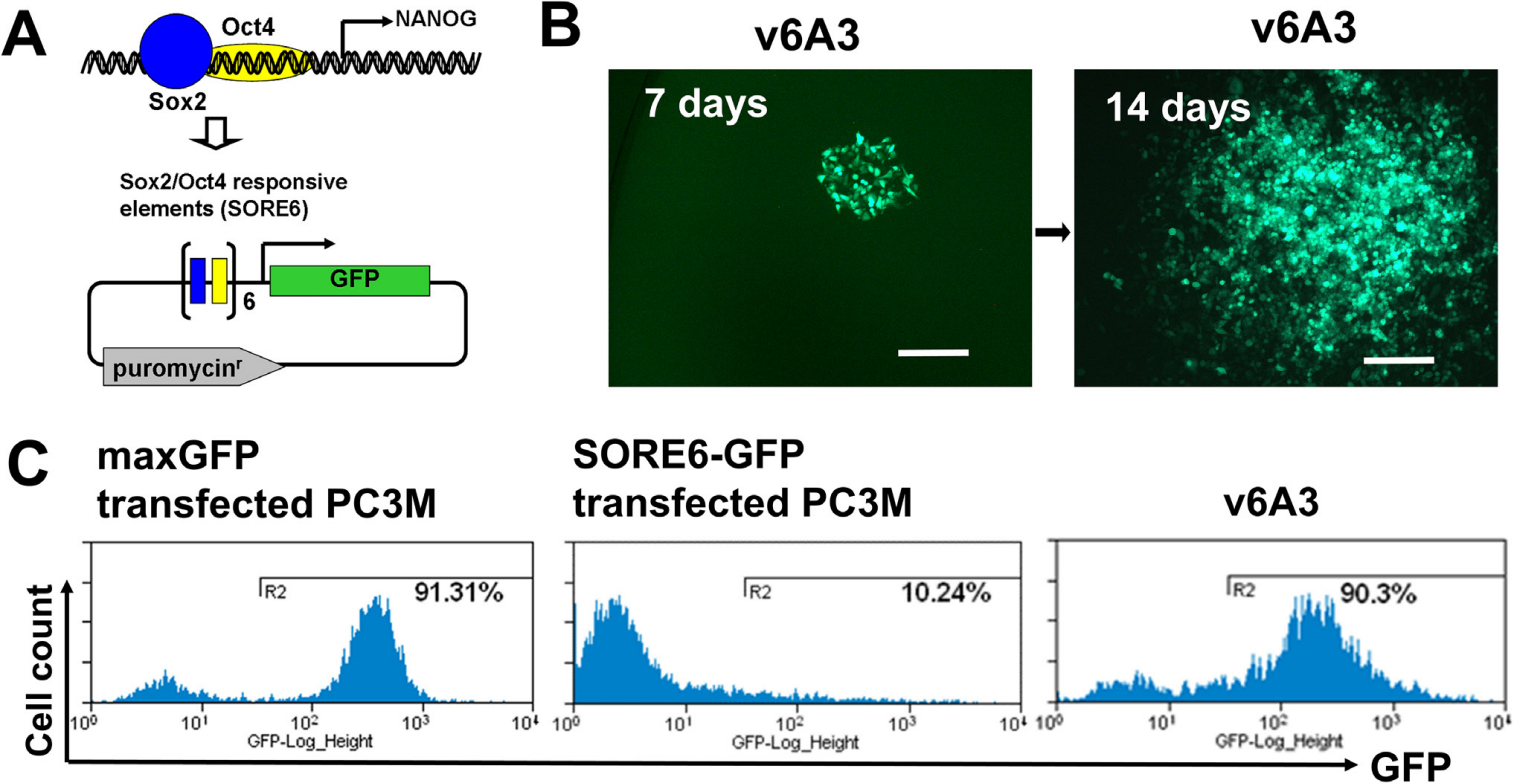
Establishment of v6A3 cell line **(A)** A schematic diagram of SORE6-GFP vector. In this vector, the GFP reporter was designed to be driven by six concatenated repeats of SOX2/OCT4 response element (SORE6) of the NANOG promoter. **(B)** The morphology of a v6A3 colony at 7 and 14 days. PC3M cells stably transfected with the SORE6-GFP construct were further treated with DOX. After being diluted and inoculated into 96 wells, one DOX resistant and GFP positive holoclone (green), v6A3, was used as an aggressive PCa cell line. Scale bar, 150 μm. **(C)** GFP positivity revealed by flow cytometry. The histogram plots represent bulk PC3M cells transiently transfected by maxGFP (∼91% GFP^+^) or SORE6-GFP (∼10% GFP+), and v6A3 cells (∼90% GFP^+^) after continuous passages for one month.

Reporter constructs driven by the NANOG promoter have been used to identify aggressive PCa cells [46]. In one study, NANOGP8-GFP, in which the 3.8-kb fragment of the NANOG genomic promotor was used to drive the expression of GFP, was constructed and applied to the study of PCa aggressive subsets [46]. However, the relatively large promoter region used in such constructs invariably contained response elements for additional transcription factors, which reduced reporter specificity. To overcome these problems, a novel CSC reporter vector, SORE6-GFP, was recently developed in the Wakefield laboratory [47]. This vector consists of a fluorescent reporter driven by six concatenated repeats of SOX2/OCT4 (SORE6) response element from the NANOG promoter (Figure 4A). Their studies demonstrated that breast cancer cell lines tagged by such reporters had the expected CSC-like aggressive properties, such as self-renewal, generation of heterogeneous offspring, high tumor- and metastasis- initiating activity, and resistance to chemotherapeutics [41]. To mark and isolate aggressive subpopulations, we transfected PC3M cells with SORE6-GFP and the control vector pmaxGFP (Lonza Inc, Allendale, NJ), respectively. Flow cytometry analysis showed that after transient transfection, only ∼10% of bulk PC3M cells were SORE6-GFP^+^ whereas pmaxGFP positivity was ∼91%, which demonstrated the high efficiency of our transfection protocol (Figure 4C). These results suggest that NANOG-expressing PCa cells are generally rare and/or NANOG expression levels in cultured bulk PCa cells are low, which is consistent with previous reports [46].

### Establishment of aggressive PCa cell model

In the original study by the Wakefield laboratory, it was found that the sorted SORE6-GFP^+^ population rapidly regenerated SORE6-GFP^-^ cell populations, which increased with passage in culture until the original equilibrium state was restored by ∼2–3 passages [41]. Such a phenotypically heterogeneous offspring was supposedly due to CSCs, which can give rise to more differentiated daughter cells. To avoid such SORE6-GFP^-^ offspring, an additional selection step might be helpful for isolating more stable and homogeneous SORE6-GFP^+^ PC3M cell clones. It was also found that on treatment with doxorubicin (DOX), extensive cell death was observed among SORE6-GFP^-^ cells while the proportion of SORE6-GFP^+^ cells in the culture increased dramatically, suggesting SORE6-GFP^+^ cells could be isolated from SORE6-GFP^-^ cells under DOX selection [41]. In addition, upregulated CD44v6 expression is known to be associated with PCa chemoresistance, suggesting CD44v6 highly expressed aggressive SORE6-GFP^+^ PC3M cells could also be enriched by DOX selection [16]. Thus in our study, the SORE6-GFP stably transfected clone PC3M1G4 was further selected by DOX to produce the v6A3 cell line (Figure 4B). As shown in Figure 4C, even after continuous passaging for one month, v6A3 cells still displayed much higher SORE6-GFP positivity (∼90%) compared to bulk transfected PC3M cells (∼10%), indicating little to no SORE6-GFP^-^ daughter cells had re-differentiated.

**Figure 5:**
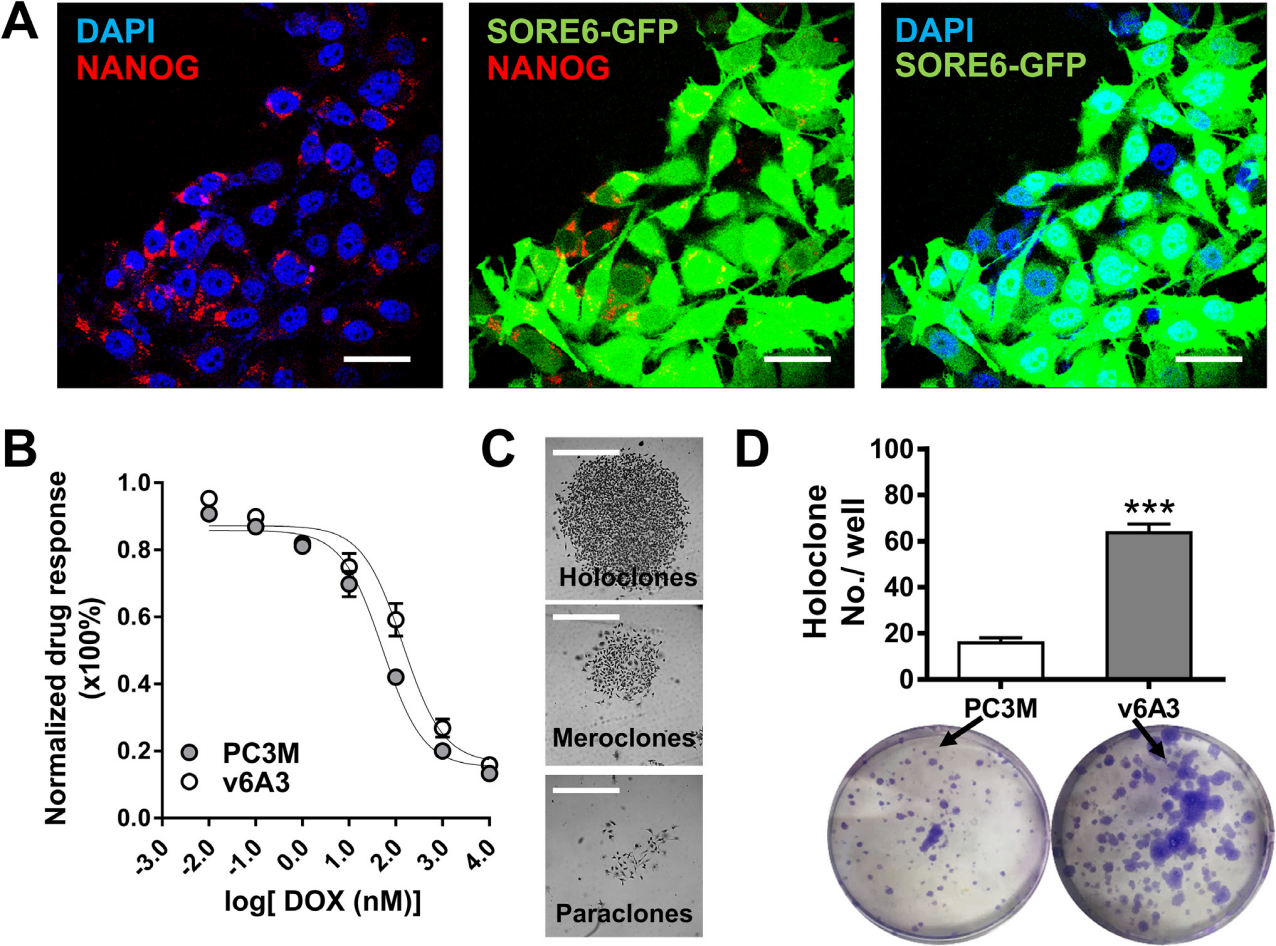
*In vitro* characterization of v6A3 cells **(A)** Confocal immunofluorescence images of NANOG (red) and concordant GFP expression (green) in v6A3 cells. The positive perinuclear NANOG staining was detected by an anti-NANOG Ab and a PE conjugated secondary Ab. Cell nuclei were stained by DAPI (blue). Scale bar, 30 μm. **(B)** The DOX resistance of v6A3. An MTT assay was used to compare the response of PC3M (gray circle) with those of v6A3 (white circle) cells to DOX. A growth inhibition curve was generated using GraphPad Prism 6.0 software. Absolute IC50 values were calculated using the log inhibitor vs. response curves for nonlinear regression. **(C)** Representative colony morphology of holoclones, meroclones and paraclones formed by v6A3. Scale bar, 300 μm. **(D)** Colony formation assay of v6A3 (gray bar) and PC3M (white bar) cells. The number of holoclones observed per well was scored to determine the efficiency of colony formation. The data presented for each group are the averages with standard deviations from triplicate samples. ***, *P* < 0.001.

SORE6-GFP^+^ breast cancer cells have previously been shown to display several expected aggressive phenotypes [16]. Thus, it was of interest to examine whether similar aggressive properties could be found in our v6A3 cells. As shown in Figure 5A, confocal immunofluorescence (IF) staining showed that positive perinuclear NANOG staining and concordant GFP expression could be observed in v6A3 cells, indicating that NANOG promoter activity and gene expression were also enriched in v6A3 cells. Because intrinsic resistance to chemotherapeutics is another typical aggressive phenotype and v6A3 cells were isolated under DOX selection, the chemotherapeutic drug sensitivities of v6A3 and PC3M cells were compared after continuous passage for one month. As shown in Figure 5B, an MTT (colorimetric 3-(4,5-dimethylthiazol-2-yl)-2,5-diphenyltetrazolium bromide) assay demonstrated that the DOX IC50 of v6A3 (140.4 nM) was higher than that of PC3M (DOX IC50: 51.59nM), suggesting v6A3 cells are more resistant to chemotherapeutic drugs (*P*< 0.05). Moreover, as shown in Figure 5C and 5D, colony forming assays demonstrated v6A3 cells generated more holoclones compared to PC3M PCa cells (∼60 holoclones per well versus ∼20 holoclones per well). An increased number of holoclones is regarded as enrichment for CSCs and the formation of holoclones has been adopted as a surrogate assay for aggressive PCa [48]. These results indicate that the v6A3 cell line contains long-term self-renewing aggressive cancer cells. Taken together, our results demonstrated that v6A3 cells represent a more aggressive subpopulation of cells isolated from the less aggressive parental PC3M cells.

### CD44v6 highly expressed PC3M cells enriched in v6A3 cells

**Figure 6:**
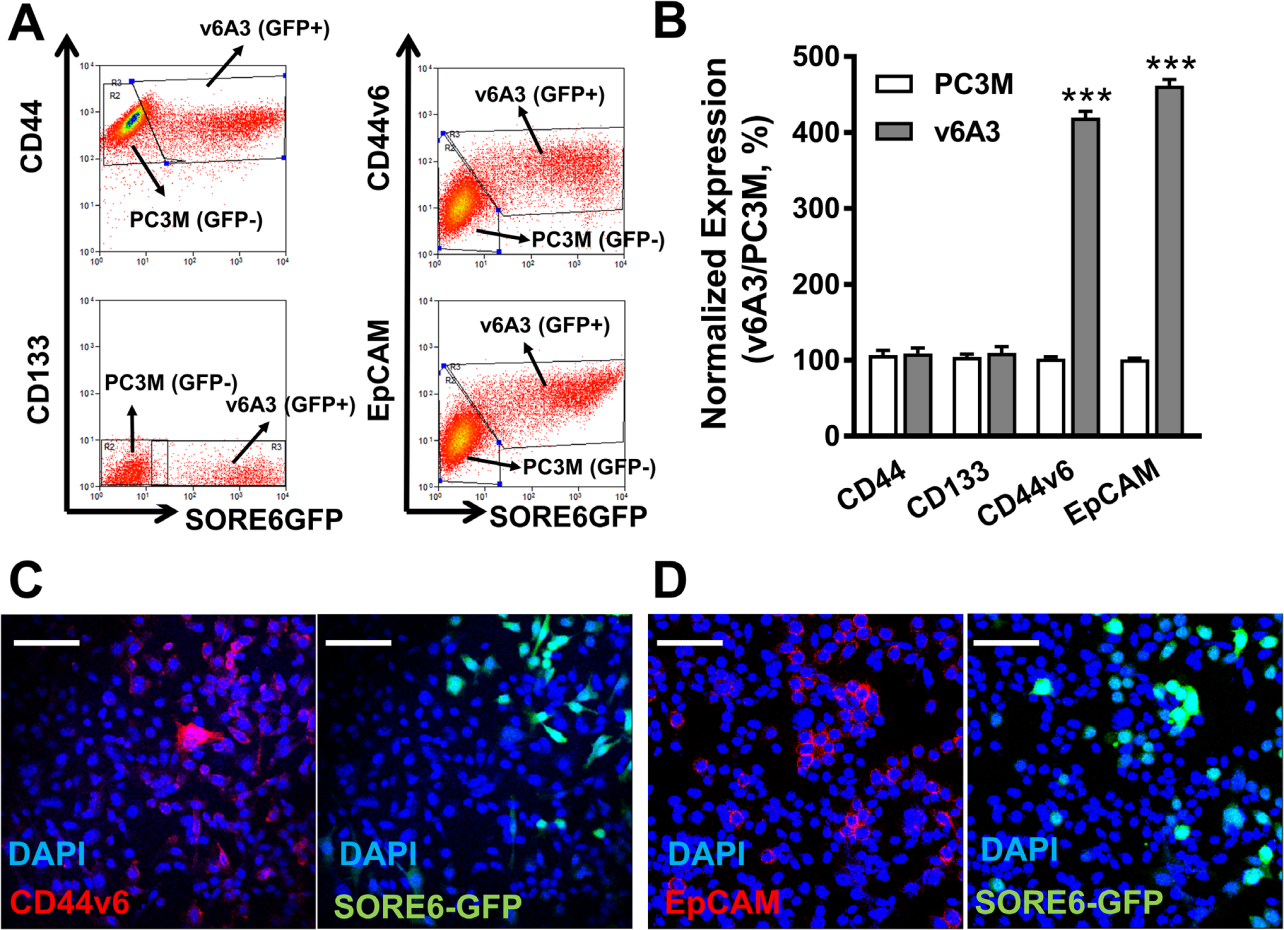
CD44v6 highly expressed PC3M subpopulations enriched in v6A3 cells **(A)** Flow cytometry analysis of the expression of CD44v6, EpCAM, CD44 and CD133 in a 1:1 mixture of PC3M (SORE6-GFP^-^) and v6A3 cells (SORE6-GFP^+^). Two gates were used based on the GFP signal (x axis) to separate PC3M and v6A3 cells. The cell bound primary Abs were detected by APC conjugated secondary Ab. The MFI of APC signal (y axis) detected from each gate was calculated to present the expression level of these four biomarkers. **(B)** The normalized expression levels of the four surface markers were calculated as the ratio of MFI of v6A3 (gray bar) to those of PC3M (white bar) cells. The data presented in each group are the averages with standard deviations from triplicate samples. ***, *P* < 0.001. Immunofluorescence confocal analysis of the expression of CD44v6 **(C)** and EpCAM **(D)** in mixed cultures of PC3M and v6A3 cells (green). The cell bound primary Abs were detected by PE conjugated secondary Ab (red). Cell nuclei were stained by DAPI (blue). Scale bar, 80 μm.

Two recent studies revealed the positive correlation between enhanced expression patterns of CD44v6 and NANOG in oropharyngeal squamous cell carcinoma (OSCC) patients [49, 50]. These discoveries prompted us to analyze the CD44v6 expression in v6A3 cells, which were tagged by the NANOG reporter vector. We also simultaneously investigated the status of three other well-recognized PCa CSC surface biomarkers, CD44, EpCAM and CD133 on v6A3 cells, considering the molecular heterogeneity within aggressive PCa populations [51]. To use PC3M cells (SORE6-GFP^-^) as an internal control, a 1:1 mixture of the same number of PC3M and v6A3 cells (SORE6-GFP^+^) was utilized for flow cytometry analyses. As shown in Figure 6A and 6B, both CD44v6 and EpCAM expression in v6A3 cells was almost fourfold higher than those in PC3M cells (*P*< 0.001), suggesting CD44v6 and EpCAM highly expressed cells have been enriched in the v6A3 cell line. In contrast, CD44 and CD133 expression levels were apparently not different between the PC3M and v6A3 cells. This finding correlates with an earlier study that found no enrichment of the CD44^+^CD24^-^ marker combination in SORE-GFP^+^ fractions of breast cancer cells [41]. Consistent with the flow cytometry data, confocal fluorescence microscopy analysis also demonstrated higher levels of expression of CD44v6 (Figure 6C) and EpCAM (Figure 6D) in v6A3 cells (SORE-GFP^+^) than in PC3M cells (SORE-GFP^-^). Taken together, these results indicate that the enriched expression of both CD44v6 and NANOG in the v6A3 cell line makes it an ideal model to investigate using PFT to detect aggressive PCa cells.

### Specificity of binding of PFT to v6A3 Cells

**Figure 7:**
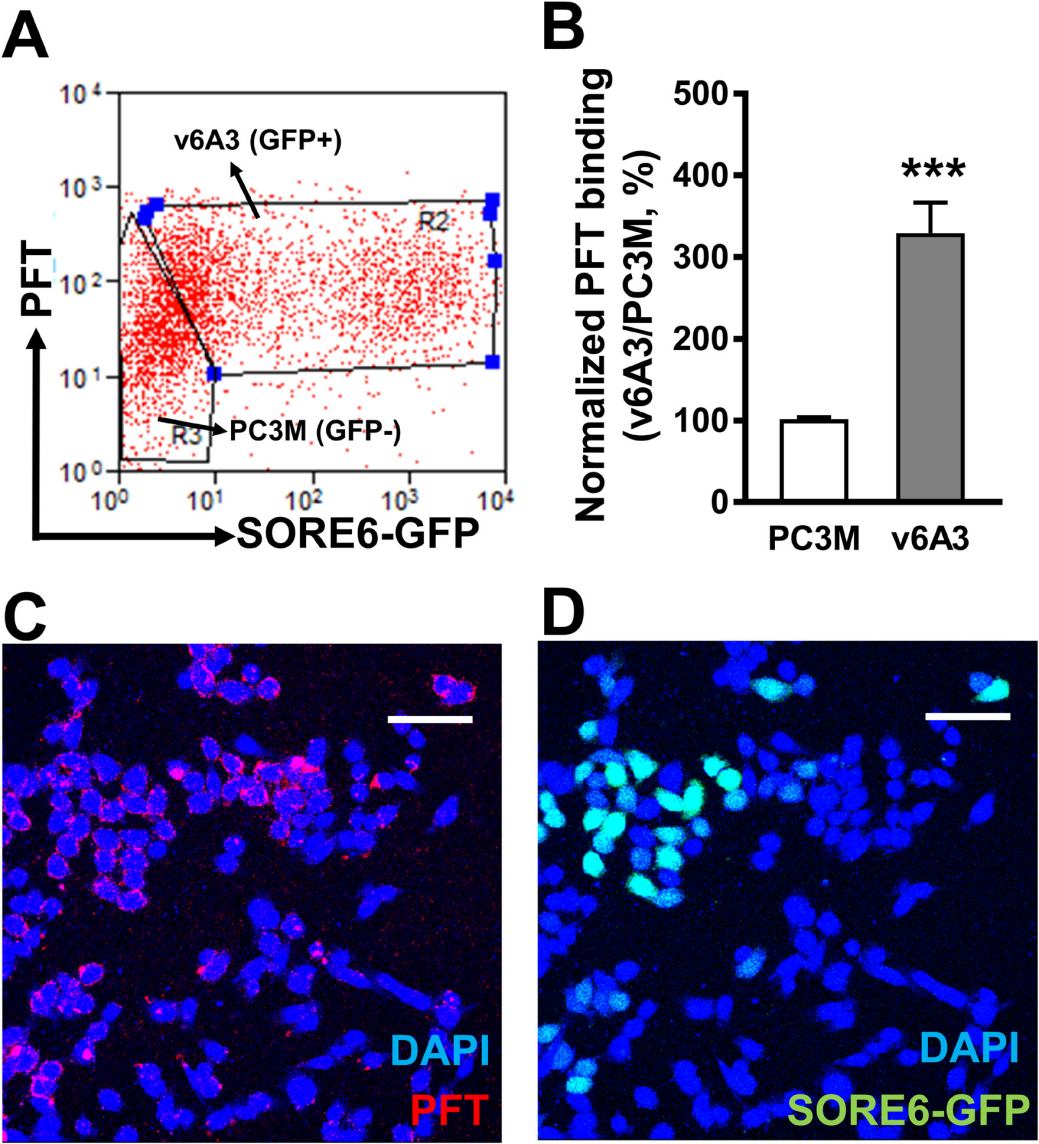
Specific binding of PFT to v6A3 cells *in vitro* **(A)** Flow cytometry analysis of PFT binding to the 1:1 mixture of PC3M (SORE6-GFP^-^) and v6A3 (SORE6-GFP^+^) cells. Two gates were set up based on the GFP signal (x axis) to separate PC3M and v6A3 cells. The cell bound biotinylated peptides were detected by APC conjugated streptavidin. The MFI of APC signal (y axis) detected from each gate was calculated to present the PFT binding level. **(B)** Normalized PFT binding levels were calculated as the ratio of MFI of v6A3 (gray bar) to those of PC3M (white bar) cells. The data presented in each group are the averages with standard deviations from triplicate samples. ***, *P* < 0.001. **(C)** Confocal fluorescence microscopy assay of PFT binding in the mixed culture of PC3M and v6A3 cells (green). Cell bound biotinylated PFT was detected by PE conjugated streptavidin (red). Cell nuclei were stained by DAPI (blue). **(D)** Confocal fluorescence microscopy assay of GFP images of paraffin-embedded v6A3 tumor xenografts from mice. The SORE6-GFP positive cells (green) were stained by FITC-conjugated anti-GFP Ab and cell nuclei were stained by DAPI (blue). Scale bar, 50 μm.

To investigate whether the PFT peptide specifically recognized CD44v6 in its natural conformation present on v6A3 cells and whether differential expression of CD44v6 between v6A3 and PC3M cells would correlate with differential PFT binding, flow cytometric and fluorescence microscopy analyses were performed on the 1:1 mixture of PC3M (SORE6-GFP^-^) and v6A3 (SORE6-GFP^+^) cells. As shown in Figure 7A and 7B, flow cytometry analysis revealed mean fluorescent intensity (MFI) values of PFT binding to PC3M and v6A3 cells of ∼35.2 and 110.7, respectively. Thus, PFT displayed more than a threefold increased binding to v6A3 compared to the parental PC3M cells (*P*< 0.001). Results of confocal fluorescence microscopy also indicated that PFT bound to GFP positive v6A3 cells preferentially over GFP negative PC3M cells (Figure 7C, 7D). Consistent with the preferential CD44v6 staining in v6A3 cells (Figure 6), these data suggest that peptide PFT binds to v6A3 cells via CD44v6 highly expressed on the cell surface membrane.

**Figure 8:**
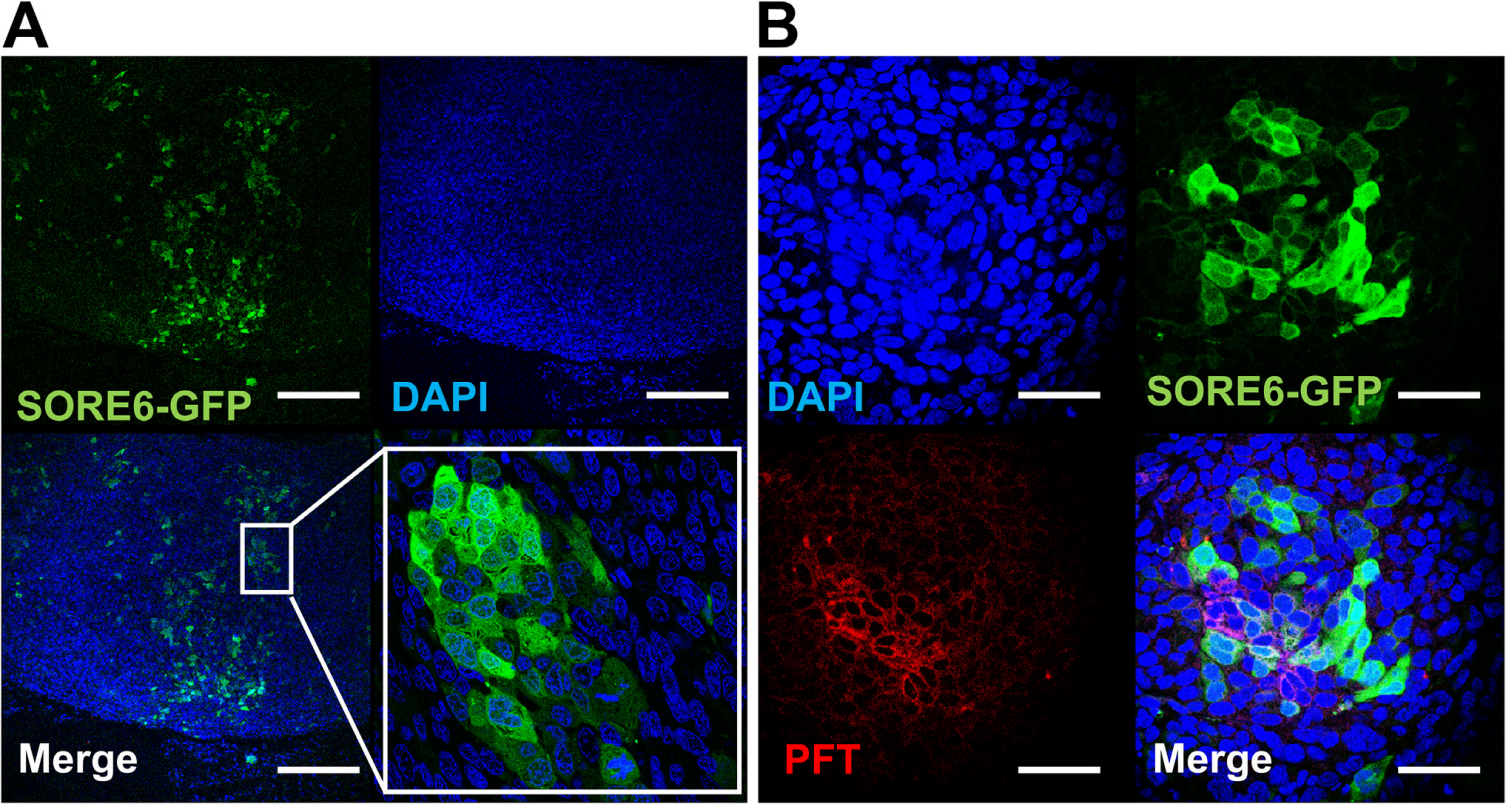
PFT staining of v6A3 xenografted tumor tissue **(A)** Confocal images of paraffin-embedded v6A3 tumor xenografts from mice. The SORE6-GFP positive cells (green) were stained by FITC-conjugated anti-GFP Ab and cell nuclei were stained by DAPI (blue). Scale bar, 200 μm. **(B)** Confocal images of PFT staining in v6A3 xenografted tumor tissue. Bound biotinylated PFT was detected by PE conjugated streptavidin (red). The cell nuclei were stained by DAPI (blue) and SORE6-GFP positive cells (green) were stained by FITC conjugated anti-GFP Ab. Scale bar, 50 μm.

To test whether the PFT peptide maintained v6A3 preferential binding in tumors, SORE6-GFP positive xenografted tumors were established in male nude mice. Although *in vitro* flow cytometric assays demonstrated that more than 90% of v6A3 cells were GFP positive, the confocal images of paraffin-embedded xenografted tumors showed that only a small portion of cells retained GFP positivity (Figure 8A). This result is consistent with the heterogeneous offspring of SORE6-GFP positive cells described in previous studies [41], suggestive of CSC-like plasticity of v6A3 cells. Because *in vivo* SORE6-GFP positive v6A3 cells still maintained the transcriptional activities of the NANOG promoter even in the complex tumor microenvironment, these cell populations are more likely to be CSC-like and/or aggressive PCa cells. Remarkably, compared to the v6A3 cells which lost SORE6-GFP expression *in vivo*, most SORE6-GFP^+^ cells in the tumor tissue were still preferentially bound by the PFT peptide (Figure 8B), illustrating a potential application of the PFT peptide in targeting aggressive PCa cells in a tumor.

### PFT binding to CD44v6 positive PCa cells in human PCa tissue specimens

**Figure 9:**
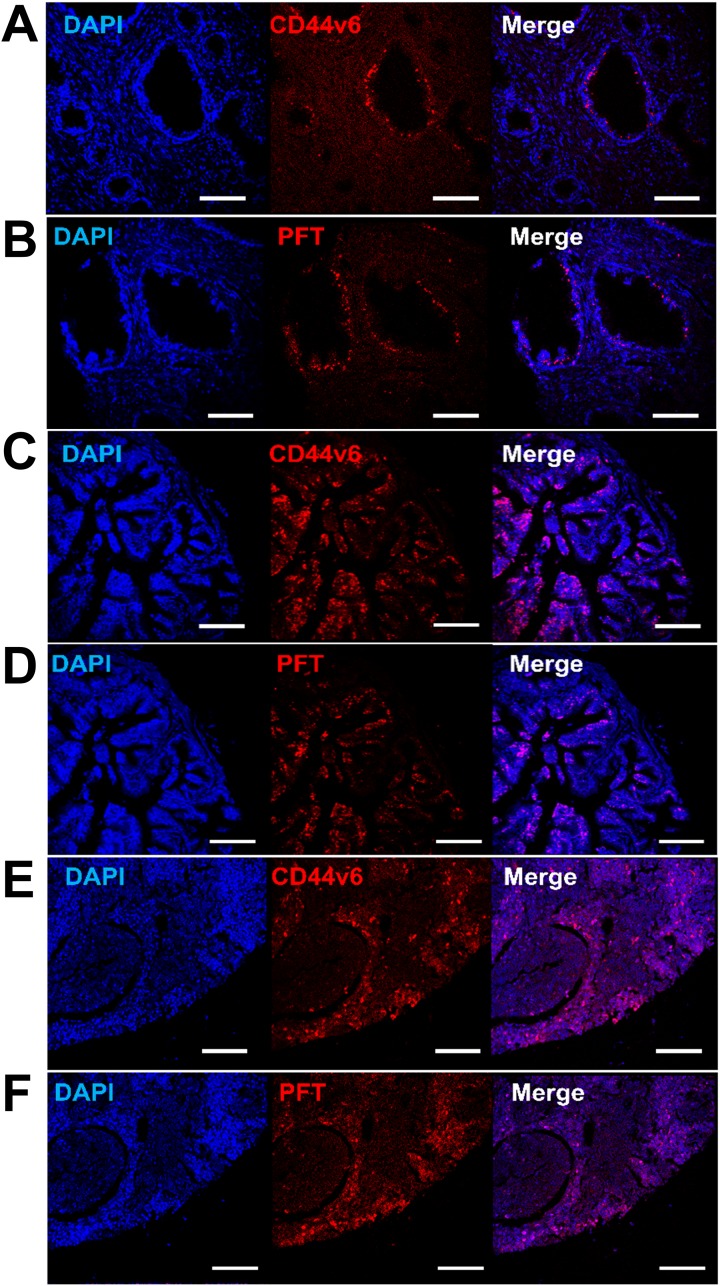
Detection of CD44v6 in PCa human TMAs using PFT peptide Immunohistofluorescence confocal images of human TMAs. Shown are representative images of CD44v6 positive (**A, C** and **E**) and PFT positive (**B, D** and **F**) staining (red) observed in human PCa samples with different staging and grading, including T2N0M0 (grade II) (A and B), T4N1M1 (grade III) (C and D), and lymph nodes metastasis (E and F). The cell nuclei were stained by DAPI (blue), the bound VFF18 was detected by PE conjugated secondary Ab (red), and the bound biotinylated PFT was detected by PE conjugated streptavidin (red). Scale bar, 150 μm.

**Figure 10:**
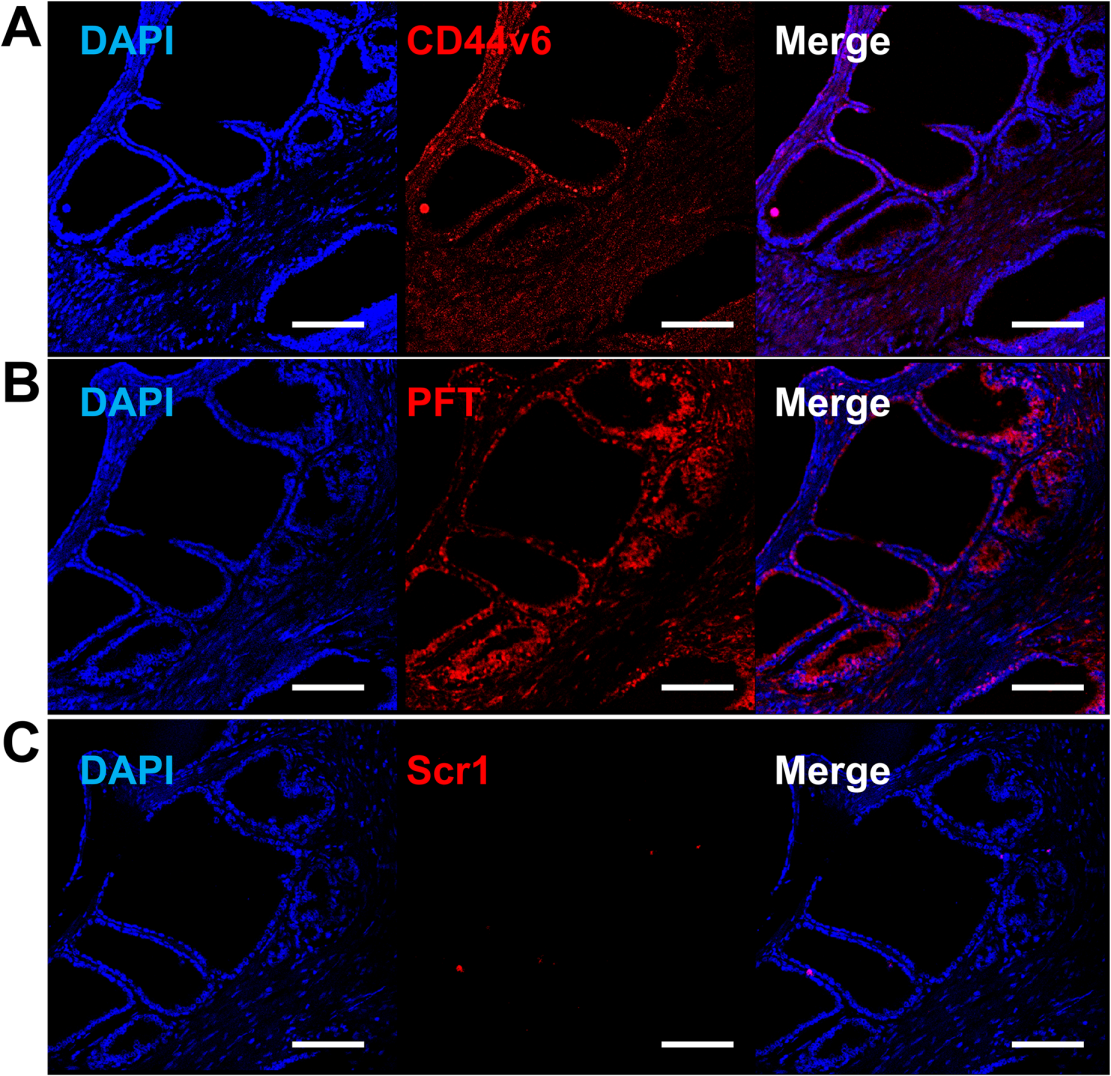
Increased PFT binding intensity over VFF18 in PCa human TMA Immunohistofluorescence confocal images of a T3N2M1 (grade III) TMA sample stained by VFF18 mAb **(A)**, PFT peptide **(B)**, and Scr1 peptide **(C)**. A difference in binding intensity was observed between samples stained by VFF18 (A) and PFT (B). The cell nuclei were stained by DAPI (blue), the bound VFF18 was detected by PE conjugated secondary Ab (red), and the bound biotinylated peptides were detected by PE conjugated streptavidin (red). Scale bar, 150 μm.

Binding of PFT peptide to SORE6-GFP^+^ cells in v6A3 tumors from xenografted mice raised the possibility that this peptide may bind to CD44v6-positive PCa cells in human tumor tissues. We tested human tissue microarrays (TMAs) that contained small representative tissue samples from multiple different cases assembled on a single histologic slide, to allow for a somewhat high throughput analysis of multiple specimens simultaneously [52]. In this study, we utilized ab178263 (Abcam, Cambridge, United Kingdom) and PR484a (US Biomax Inc., Rockville, MD) human PCa tissue arrays containing 60 prostate tumor samples and 36 normal and reactive (hyperplasia and inflammatory) prostate tissue samples with Gleason grading and TNM staging (T, primary tumor; N, regional lymph nodes; M, distant metastasis) data [53, 54]. As shown in Figure 9 and 10, immunohistofluorescence confocal analyses demonstrated that CD44v6 was highly expressed in PCa samples, including those of different stage (Figure 9A and 9C) and lymph node metastases (Figure 9E). PFT was highly accurate for the detection of CD44v6 positive samples of variable stage and grade (Figure 9B, 9D and 9F). These results suggested that PFT peptide-mediated binding to CD44v6 could be used as a highly accurate means to diagnosis aggressive PCa lesions, even in early stages (Figure 9D) and metastatic tumors (Figure 9E). Furthermore, PFT staining positivity was found to be significantly higher in late stage (T3-T4) (*P*=0.0110), metastatic (M1) (*P*=0.0060), and higher grade (III-V) samples (*P*=0.0012) (Table 2). It was also observed that peptide PFT had comparable if not greater staining intensity compared to mAb VFF18 in most malignant tissues (Figure 9) but not in normal or reactive (hyperplasia and inflammatory) tissues (Table 2). In some samples, such as a T3N2M1 (grade III) sample shown in Figure 10, PFT (Figure 10B) staining intensity was even higher than those of mAb VFF18 (Figure 10A). Importantly, no staining was observed for a scrambled version of PFT to PCa tissue (Figure 10C), evidence that PFT staining is CD44v6-specific.

**Table 2:** PFT staining positivity and clinicopathological variables

Variable	Samples	PFT staining positivity (%)	*P*-value (χ2 test)^a^
Normal	6	0	
Benign	4	0	
Hyperplasia	22	0	
Inflammatory	4	0	
T (primary tumor) stage			
T1-2	44	61.4(27/44)	0.0110^b^
T3-4	12	100 (12/12)	
N(regional lymph nodes) stage			
N0	48	64.6 (31/48)	0.0901
N1-2	8	100 (8/8)	
M (distant metastasis) stage			
M0	46	63.0 (29/46)	0.0060^b^
M1	14	100 (14/14)	
Grade			
I-II	16	37.5 (6/16)	0.0012^b^
III-V	38	84.2 (32/38)	

^a^ Statistical comparisons were made between samples of T1-2 with T3-4, N0 with N1-2, M0 with M1, and Grade I-II with Grade III-V.

^b^ *P*<0.01.

## DISCUSSION

Affinity selection of phage displaying peptides typically involves passing the phage library over desired target molecules, such as protein biomarkers of cancer [27, 28]. In this study, we aimed to obtain peptides specific to the v6 region of human CD44v6, a putative aggressive cancer and/or CSC biomarker. However, the v6 region contains only 45 amino acids while the CD44v6-ECD consists of 290 amino acids [10]. Thus, affinity selection against the CD44v6-ECD or entire protein would prove challenging for the direct identification of v6 specific-binding peptides. VFF18 is a murine mAb generated by immunizing BALB/c mice with a glutathione S-transferase (GST) fusion protein containing the human CD44 variant portion v3-v10. Epitope mapping results showed that peptide WFGNRWHEGYR, corresponding to amino acids 19-29 of the v6 domain, represented the minimum sequence required for high-affinity binding of VFF18 [55]. Furthermore, it was shown that a v6 peptide derivative, KEQWFGNRWHEGYR, retained function in that it inhibited Met signaling [36]. Therefore, we used this peptide to capture v6-specific phage clones. Phage libraries displaying linear peptides and disulfide-constrained peptides were subjected to three rounds of selection based on their ability to bind the peptide. The fUSE5/15mer phage displayed peptide PFT was identified and it demonstrated binding to both recombinant- and PCa cell- associated CD44v6 once chemically synthesized as a linear peptide. It is well known that inherited and acquired changes in pre-mRNA splicing play a significant role in human disease development and many cancer-associated genes are regulated by alternative splicing, such as CD44, the Wilms' tumor gene WT1, BRCA1, MDM2, FGFR and kallikrein family members [56, 57]. Most of the classic protein cancer biomarkers, such as epidermal growth factor receptor (EGFR), have been found on non-malignant cells, albeit usually at lower levels than cancer cells [58]. In contrast, since alternative splicing of protein pre-mRNA in cancer cells often results in new altered protein domains not produced in normal cells, the peptide epitope of these cancer-specific domains could be used as a more specific biomarker to discriminate between tumor and normal tissues [56]. Our study presented here provides evidence that peptides representing alternative splicing domains could be used for phage display selection of peptides that target splice variant biomarkers of cancer.

NANOG is a member of the homeobox family of DNA binding transcription factors and plays an important role in the maintenance of pluripotency and self-renewal of human ESCs [59]. Several studies have shown that the expression level of NANOG is high in in a variety of cancers, including those of the prostate, and low or absent in normal tissues [43, 44]. Of interest, NANOG expression has been found to be localized more frequently at the fringe of invasive PCa tissues, and correlated significantly with various aggressive cell behaviors including TNM classification and clinical stage [45]. Moreover, extensive loss-of-function analyses revealed that RNAi-mediated NANOG knockdown inhibited xenograft tumor development in several prostate cancer cell lines [44]. It was further shown that NANOG overexpression fostered the expression of other molecules, including BCL-2, IGFBP-5 and CXCR4, which may contribute to ADT resistance in PCa [46]. These results pointed to a vital role of NANOG in PCa aggressiveness. Being prompted by these findings, here we developed a SORE6-GFP stable transfected and DOX selected PC3M cell line, v6A3, as an aggressive PCa model system. Our study demonstrated that v6A3 cells have the expected aggressive PCa phenotype including NANOG expression, enhanced clonogenicity, resistance to chemotherapeutics and generation of heterogeneous offspring, suggesting this SORE6-GFP reporter system could be used to mark and isolate aggressive subsets from established cancer cell lines.

The expression pattern of CD44v6, NANOG and its correlation with different anatomical subsites, loco- regional aggressiveness and recurrence of OSCC were recently investigated by Rawal et al [49, 50]. Quantitative gene expression analysis of circulating tumor cells from OSCC patients demonstrated a significant upregulation in gene expression of CD44v6 and NANOG. Moreover, significantly higher co-expression of CD44v6 and NANOG was found in late stage and loco-regionally aggressive patients compared to early stage and non-aggressive OSCC counterparts. Significantly increased expression of both CD44v6 and NANOG in recurrent OSCC cases compared to non-recurrent cases was also noted, indicating a possible role of these markers as secondary malignancy risk predictors [49, 50]. Both NANOG and CD44 expression levels have been shown to correlate in human PC3 prostate cancer cells [60]. However, to date, the link between CD44v6 and NANOG in PCa remains poorly understood. In the future it will be interesting to determine the correlation of CD44v6 and NANOG in the chemoresistant A3 prostate cancer cells and in the progression and invasion of prostate cancer. NANOG has previously been shown to be involved in tumor progression and castration resistance by binding to the androgen receptor/FoxA1 signaling complex [46]. Thus, the possibility that CD44v6 is involved in this signaling pathway may also be explored. In the present study, a correlation was found between SORE6-GFP expression, CD44v6 expression, and PFT binding in v6A3 cells, both *in vitro* and *ex vivo*. To the best of our knowledge, this is the first study establishing the importance of CD44v6 and NANOG in aggressive PCa cells. It will be interesting in the future to examine the correlation of CD44v6 and NANOG in tumor invasion and progression of PCa tumors.

The TMA represents a high-throughput technology for the assessment of histology-based laboratory tests, such as immunohistochemistry. In addition, this technology has served as an excellent preclinical translation platform for the assessment of novel molecular biomarkers and new diagnostic tools in human cancers [52]. In our study, TMA immunohistofluorescence assays demonstrated the accuracy of PFT for detecting CD44v6 positive PCa patient tissues. Additionally, there was significant correlation between PFT staining positivity and aggressive PCa characteristics, including late TNM stage, higher clinicopathological grade and distance metastasis. Thus, PFT could possibly play a role in detecting aggressive primary PCa or metastasis in advanced PCa disease. Taking advantage of PFT’s smaller size and better tumor penetration might allow for detection of CD44v6 positive lesions which are unable to be recognized by mAb staining. In addition, for the vast majority of aggressive PCa, surgical resection is the treatment choice whenever feasible. Considering its capacities to localize and image primary and metastatic PCa cells, PFT might be able to be developed into an intraoperative real-time probe to reduce more invasive surgical approaches that often cause damage to surrounding nerves, bladder, or sphincter [61, 62]. A drawback in this approach is that peptides including PFT often have high nanomolar affinities making their *in vivo* application challenging. Improved affinity of PFT could be achieved by affinity maturation phage display approaches [63] or by engineering PFT into scaffolds such as cyclotides [64] or molecular carriers such as silica nanoparticles with optimal *in vivo* biodistribution properties for targeted imaging or therapy [65]. A future goal of this work will be to examine if PFT peptide or PFT peptide scaffolds or nanoparticles can be utilized *in vivo* to systematically deliver tumors therapeutic and diagnostic molecules to CD44v6 overexpressing aggressive PCa cells in animal models of PCa as well as patients.

## MATERIALS AND METHODS

### Materials

Unless otherwise indicated, cell culture reagents were purchased from Invitrogen (Carlsbad, CA), antibodies were purchased from Thermofisher (Waltham, MA), molecular biology reagents were purchased from NewEngland Biolabs (MA, USA), and other chemicals were purchased from Sigma Chemical Co. (St. Louis, MO).

### Cell lines and culture

The human prostate cancer (PCa) cell line PC3M, kindly provided by Dr. Avraham Raz (Wayne State University, Detroit, MI), was grown to confluence in RPMI 1640 medium (Gibco BRL, Grand Island, NY) supplemented with 2mM L-glutamine, 10% fetal bovine serum (FBS), non-essential amino acids and 48 mg/ml gentamicin at 37°C in 5% CO_2_. The human PCa cell line PC3 (ATCC^®^ CRL-1435™) and human ovarian cancer cell line SKOV3 (ATCC^®^ HTB-77^™^) were grown in RPMI 1640 supplemented with 10% FBS, 2 mM L-glutamine, and 48 mg/ml gentamicin at 37°C in 5% CO_2_. MDA PCa 2b (ATCC^®^ CRL-2422™) human PCa cells were grown in BRFF-HPC1 media (AthenaES, Baltimore, MD) supplemented with 20% FBS, 2 mM L-glutamine, and 48 mg/ml gentamicin at 37°C in 5% CO_2_ on poly-lysine coated plates. The Chinese hamster ovary epithelial cell line CHO-K1 (ATCC^®^ CCL-61™) were grown in F-12K Medium (Gibco BRL, Grand Island, NY) supplemented with 10% FBS, 2 mM L-glutamine, and 48 mg/ml gentamicin at 37°C in 5% CO_2_. Subculturing of the cell lines was performed using standard trypsinization procedures.

### Peptide synthesis and purification

All peptides were synthesized using an Advanced Chem Tech 396 multiple peptide synthesizer (Advanced ChemTech, Louisville, KY) using solid-phase Fmoc chemistry. A linker amino acid sequence of Gly-Ser-Gly (GSG) was inserted between the biotin and the NH_2_ terminus of the peptide to minimize any steric or chemical effects. Peptides were purified by reverse-phase high-pressure liquid chromatography (RP-HPLC) on a C_18_ column (218TP54, Vydac, Hesparia, CA), lyophilized, and stored at −20°C. Purity of the peptides was greater than 90% as assessed by HPLC. Identities of the peptides were confirmed by electrospray ionization mass spectrometry (Mass Consortium, San Diego, CA) at the Structure Core of University of Missouri, Columbia.

### Affinity selection

Phage libraries displaying linear peptides (fUSE5/15mer, gift of Dr. George Smith, University of Missouri-Columbia) or disulfide-constrained peptide libraries (M13C7C, New England Biolabs, Beverly, MA, USA) were used for three rounds of affinity selection. The affinity selection of fUSE5/15mer was performed following the protocol described by George Smith (Scott and Smith, 1990) as detailed on the website http:/www.biosci.missouri.edu/SmithGP. The M13C7C library was selected as described by the manufacturers protocol for the PhD phage system. The biotinylated v6 region peptide KEQWFGNRWHEGYR and the control peptide [66], RNVPPIFNDVYWIAF (1.25 nmol/100 μl/well) were incubated with BSA-blocked streptavidin-coated strip wells (Thermofisher, Waltham, MA) at 4°C for 2 h. Next, 5x10^8^ virons of fUSE5 phage library or 2×10^10^ pfu of M13C7C phage library were incubated with immobilized v6 region peptide per well for 4 h at room temperature (RT). Plates were washed 10 times in the 1^st^ round and 20 times for the 2^nd^ and 3^rd^ rounds, with Tris-buffered saline (TBS) containing Tween 20 (TBST) to remove unbound phage. Tween 20 concentrations increased stepwise (0.1%, 0.3%, 0.5%) in the 3 rounds of affinity selection. Bound phage were eluted using 0.2 M glycine–HCl pH 2.2, 1 mg/ml BSA and were neutralized with 1 M Tris–HCl pH 9.1. After the 1^st^ and the 2^nd^ rounds, the eluate of the bound phage was propagated in the *Escherichia coli (E. coli)* and used as input for the next round of selection. Individual phage clones displaying putative human CD44v6-binding peptides were prepared and their concentrations were determined by measurement of virions per volume for fUSE5/15mer and plaque forming units (pfu) for M13C7C. Individual clones were characterized by DNA sequencing after the third round of selection.

### Phage ELISA

To test for phage binding to v6 region peptide, phage were diluted in TBS (5x10^10^ virons of fUSE5 phage or 2×10^12^ pfu of M13C7C phage) and incubated with immobilized v6 region peptide (0.5 μg/well) for 2 h at RT. The wells were then washed five times with TBST and probed with a mouse monoclonal M13/fd/F1 filamentous phage antibody (0.2 μg/ml in TBST, Fitzgerald, MA) for 1 h at RT. After washing with TBST buffer five times, an anti-mouse IgG-horseradish peroxidase (HRP) conjugate was added and incubated for 1 h, followed by a reaction with fast O-phenylenediamine dihydrochloride (Sigma-Aldrich, St. Louis, MO). After 30 min, the reaction was terminated by the addition of 50 μl of 3 M HCl solution to each well and the optical densities were measured with a μQuant Universal Microplate Spectrophotometer (Bio-Tek Instruments, Winooski, VT) at 490 nm.

### Peptide-Peptide ELISA

An ELISA was performed to analyze the binding of synthesized biotinylated peptides to biotinylated v6 and control peptides used in phage display selection. Streptavidin coated high capacity plates (Thermofisher, Waltham, MA) were incubated with 100 μL of v6 and control peptides in wash buffer (25 mM Tris pH 7.2, 150 mM NaCl, 0.1% BSA, 0.05% Tween-20) at 2 μM for 1 h at RT with shaking. After washing plates 3 times with wash buffer, 100 μL of biotin at 10 μM was then added to each well and incubated for 30 min at RT with shaking. After washing 3 times with wash buffer, plates were incubated with 100 μL of peptides at 10 μM in wash buffer for 2 h at RT with shaking. After washing 5 times with wash buffer, plates were incubated with 100 μL of HRP-conjugated streptavidin (1:2000 diluted in wash buffer) for 1 h at RT with shaking. After washing five times with wash buffer, the Sigma fast O-phenylenediamine dihydrochloride tablets (Sigma-Aldrich, St. Louis, MO) were used as substrates and the absorbance was measured at 490 nm using an endpoint assay on a μQuant Universal Microplate Spectrophotometer (Bio-Tek Instruments, Winooski, VT).

### Peptide-Protein ELISA

An ELISA was performed to analyze the binding of synthesized biotinylated peptides to recombinant CD44-Fc or CD44v6-Fc. Briefly, 100 μl of recombinant protein at 1 μg/ml in 0.05 M carbonate/bicarbonate coating buffer (pH 9.6) was added to each well of a 96-well microtiter plate and coated for 24 h at 4°C. The plates were blocked with 1% BSA in PBST buffer (0.05% Tween 20 in PBS) and then incubated with 100 μl of peptides or antibodies at various concentrations in 0.5% BSA in PBST for 2 h at RT. After washing extensively for five times with PBST buffer, the plates were incubated with 100 μl of HRP-conjugated streptavidin (1:2000 diluted with 0.5% BSA in PBST buffer) for 1 h at RT. After washing five times with PBST buffer, the Sigma fast O-phenylenediamine dihydrochloride tablets (Sigma-Aldrich, St. Louis, MO) were used as substrates and the absorbance was measured at 490 nm using an endpoint assay on a μQuant Universal Microplate Spectrophotometer (Bio-Tek Instruments, Winooski, VT).

### Production and purification of CD44-Fc and CD44v6-Fc

For binding analysis, the ECD of CD44 and CD44v6 protein were expressed as Fc-tagged proteins (Figure 2A). Exon numbering was performed according to the study of Screaton et al. The ECD of CD44 was engineered as an N-terminal constant sequence (exons 1–5) followed by a nonvariable stretch at the C-terminus (exons 15–17) (Figure 2A). The ECD of CD44v6 was engineered as a protein with the v6 variable region sequence (exon 10) flanked by the N- and C- terminal constant region of CD44 (Figure 2A). Total RNA extracted from human ovarian cancer cell line SKOV3 was used as template for reverse transcription and PCR amplification. PCR primers were designed based on the published sequences of CD44 isoforms in UniProt (entry P16070). As shown in Table 3, primer p44e1F*Eco*RI and p44e5R were used to amplify exons 1–5 to produce fragment CD44N. Primer p44e1F*Eco*RI and p44v6R were used to amplify exons 1–5 followed by exon 10 to produce fragment CD44Nv6. Primer p44e15F and p44e17R*Bgl*II were used to amplify exons 15–17 to produce fragment CD44C. Because both primers p44e5R and p44v6R were designed to carry a 5' overhang complementary to the 5' end of primer p44e15F (as shown underlined), the fragment CD44N or CD44Nv6 were fused to fragment CD44C by overlap extension PCR to produce fragment CD44-ECD and CD44v6-ECD, respectively. These two ECD fragments were then cloned into the *Eco*RI / *Bgl*II site of vector pFUSE-hIgG1-Fc2 (InvivoGen, San Diego, CA) to produce the human Fc fusion construction pCD44Fc and pCD44v6Fc individually. Restriction enzyme analysis and DNA sequencing were used to identify the constructs containing the correct expression cassette. Following transient transfection into CHO-K1 cells, individual clones arising from single cells were isolated using serial limiting dilution and selected in medium containing Zeocin (100 μg/ml). The supernatant from each clone was assayed for the expression of recombinant protein by enzyme-linked ELISA using an anti-IgG1Fc mouse mAb (Santa Cruz Biotechnology, Santa Cruz, CA). The highest expressing clone of CD44-Fc and CD44v6-Fc was chosen for large-scale cultivation in medium containing 4% low IgG FBS (Thermofisher, Waltham, MA) and purified by protein A (GenScript, Piscataway, NJ) chromatography in accordance with the manufacturer’s protocol. Following purification, protein concentration was determined using a Bradford assay (Bio-Rad Laboratories, Hercules, CA, USA) and the final protein products were evaluated by SDS-PAGE and immunoblotting.

**Table 3:** Primers used for CD44v6-Fc and CD44-Fc construction

Primers	Sequences
p44e1FEcoRI	5'-ACGAATTCGCAGATCGATTTGAATATAACCTGCC-3'
p44e5R	5'-GGAATGTGTCTTGGTCGATGGTAGCAGGGATTC-3'
p44v6R	5'-GGAATGTGTCTTGGTCTGCAGCTGTCCCTGTTGTCG-3'
p44e15F	5'-GACCAAGACACATTCCACCCCAGTG-3'
p44e17RBglII	5'-GTCAGATCTATCATCATCATCTTTTTCTGGAATTTGGGGTGTCC-3'

### SDS-PAGE and immunoblotting

Cells were collected and lysed in hypotonic buffer with nonionic detergent (50 mM Tris-HCl, pH 7.5, 150 mM NaCl, 0.5% NP-40, 50 mM NaF with 0.5 mM phenylmethylsulfonyl fluoride), incubated on ice for 15 min and cleared by centrifugation at 10,000 x g at 4°C for 10 min. Protein concentration was determined using a Bradford assay (Bio-Rad Laboratories, Hercules, CA, USA). Equal amounts of protein were mixed with reducing Laemmli loading buffer, boiled and electrophoresed on NuPAGE 4-12% Bis-Tris Gels (Invitrogen, Carlsbad, CA), then transferred electrophoretically to nitrocellulose membranes in Tris-glycine buffer. The membranes were blocked with 5% nonfat dry milk in TBST (with 0.05% Tween 20) for 1 h at RT, and then incubated with 1 μg/ml primary antibodies at 4°C overnight. After washing five times (5 min each wash) with TBST buffer, the membranes were incubated with HRP-conjugated goat anti-mouse IgG (1:10,000 dilution, R&D Systems, Minneapolis, MN) for 1 h at RT. The reactions were detected by using an ECL Western blot detection kit (Thermofisher, Waltham, MA) and the images were recorded by VersaDOC imaging system (BioRad, Hercules, CA).

### Fluorescence microscopy

Binding of biotinylated peptides or antibodies to human cancer cells was evaluated using fluorescence microscopy. Cells were grown on microscope chamber slides (Lab-Tek, Rochester, NY) and fixed with 4% paraformaldehyde for 15 min, followed by blocking with 5% BSA in PBS for 2 h. The cells were then washed three times with PBST and incubated with 50 μM peptides or 3 μg/ml primary mouse mAbs (Thermofisher, Waltham, MA) at RT for 1 h. Next, fixed cells were washed extensively with PBST and incubated with 0.5 μg/ml PE-labeled streptavidin or PE-labeled goat anti-mouse IgG (Thermofisher, Waltham, MA) for 1 h at RT in the dark and washed three times with PBST. Cell nuclei were stained by 4,6-diamidino-2-phenylindole (DAPI) in mounting medium (1:500) (Invitrogen, Carlsbad, CA) and slides were viewed and the images were recorded by a Zeiss LSM 5 LIVE line-scanning confocal microscope (Carl Zeiss, Oberkochen, Germany) at Harry S. Truman Veterans Memorial Hospital.

### Flow cytometry analysis

Cultured cells were removed from flasks using cell dissociation buffer (Thermofisher, Waltham, MA) and resuspended in ice cold flow cytometry buffer (Dulbecco PBS, 1% BSA, 0.1% NaN3, 5 mM EDTA) at 100 μl/10^6^ cells. Next, 50 μM peptides or 3 μg/ml primary mouse mAbs diluted in flow cytometry buffer were incubated with cells at 4°C with gentle shaking for 1 h. After washing three times with flow cytometry buffer to remove unbound peptides or antibodies, cells were incubated with 0.5 μg/ml APC-conjugated streptavidin or secondary antibodies (Thermofisher, Waltham, MA) at 4°C for 45 min. After washing three times, cells were kept at 4°C until analysis using a BD CyAn ADP flow cytometer (BD Biosciences, San Jose, CA) and Summit 5.2 software (BD Biosciences, San Jose, CA) at the cell and immunology core in University of Missouri, Columbia.

### Stable transfection of PC3M cells using SORE6-GFP

Transfection was performed with polyethyleneimide (PEI) (Polysciences, Warrington, PA) and SORE6-GFP plasmid-DNA as described previously [67]. Individual stable transfection clones were isolated using serial limiting dilution and selected in medium containing puromycin (2μg/ml). The GFP positive cell clones were identified and observed with an epifluorescent Nikon T1-SM inverted microscope (Nikon, Melville, NY). After two weeks, the four fastest growing GFP positive clones (PC3M1A4, PC3M1G4, PC3M3F9 and PC3M3H11) were tested by flow cytometry and PC3M1G4 was picked for further study because it had the highest GFP and CD44v6 expression (data not shown). To enrich CD44v6 overexpressed SORE6-GFP positive PC3M cells, PC3M1G4 was cultured *in vitro* with continuous exposure to 25 nM DOX (https://dtp.cancer.gov/organization/dscb/) for 7 days, followed by a 14 day recovery period in the absence of drug, as described previously [68]. After three rounds of treatment, single PC3M cells were transferred into the single well of 96-well plates until drug-resistant clones formed. That fastest growing clone, PC3M1G4A3 (hereinafter referred to as v6A3 cells) was selected to analyze its aggressive phenotype.

### Examination of chemodrug resistance

To determine the dose response of drug-resistant PC3M1G4 cell lines, PC3M and v6A3 cells were seeded in triplicate in 96-well plates at 5000 cells per well and incubated for 24 h. A range of concentrations (1000, 100, 10, 1, 0.1, 0.01, 0.001, 0.0001 nM) of DOX in fresh medium were added to the cells. Control cells were treated with appropriate volumes of fresh medium. After 48 h, 20 μl of MTT (3-(4,5-dimethylthiazol-2-yl)-2,5-diphenyltetrazolium bromide) (5 mg/ ml) (Sigma-Aldrich, St. Louis, MO) was added to each well, followed by incubation at 37°C in 5% CO_2_ for 4 h. Subsequently, 100 μl of DMSO (Sigma-Aldrich, St. Louis, MO) was added and the plate was shaken for 20 min at RT to dissolve the formazan crystals. The optical density (absorbance units) was read at a wavelength of 562 nm on a μQuant Universal Microplate Spectrophotometer (Bio-Tek Instruments, Winooski, VT). The growth inhibition curve was generated using GraphPad Prism 6.0 software (GraphPad Software Inc., San Diego, CA). Absolute IC50 values were calculated using the log inhibitor vs. response curves for nonlinear regression.

### Colony formation assay

Briefly, v6A3 cells or PC3M cells were seeded in 6-well plates at low density (∼1,000 cells per well) and cultured for 6 days. The plates were then washed with PBS and stained with crystal violet [48]. The images of each well were scanned, and the individual clone types were identified. Holoclones were round in shape, and the cells comprising them were tightly packed and relatively small in size. Paraclones were irregular in shape and comprised of loosely packed cells, whereas the morphology of meroclones was intermediate to that of holoclones and paraclones (Figure 5C) [48]. The number of holoclones per well was scored to determine the efficiency of colony formation.

### Establishment of v6A3 xenografted tumors

Three male nude (*nu*/ *nu*) mice (Harlan Sprague Dawley Inc, Indianapolis, IN) were subcutaneously inoculated with 1×10^7^ v6A3 cells and the tumors were allowed to grow until tumor size ranged from 0.2 to 0.5 cm in diameter. Animals were sacrificed and the excised tumors were fixed and paraffin-embedded for immunohistochemical studies. All animal studies were conducted according to NIH Guidelines for the Care and Use of Laboratory Animals and the Policy and Procedures for Animal Research of the Harry S. Truman Veterans Memorial Hospital and housed in pathogen-free conditions.

### Immunohistofluorescence assay

Formalin fixed and paraffin embedded PCa xenografted tumor specimens or patient tissue microarray ab178263 (Abcam, Cambridge, United Kingdom) and PR484a (US Biomax Inc., Rockville, MD) were deparaffinized in three changes of xylene for 5 min each and then rehydrated to distilled water using graded alcohols. The epitope was recovered by steaming the slides in R-Universal Epitope Recovery Buffer (Electron Microscopy Sciences, Hatfield, PA) for 15 min, followed by transferring the slides into PBST buffer for 5 min. After blocking with 10% horse serum at RT for 1 h, endogenous biotin or mouse IgG was blocked with Endogenous Biotin-Blocking Kit (Molecular Probes, Eugene, Oregon) or unconjugated F(ab) fragment of anti-mouse IgG, separately (Abcam, Cambridge, United Kingdom). After extensive washing with PBST, the slides were stained with 50 μM peptides or 3 μg/ml primary mouse mAbs (Thermofisher, Waltham, MA) at 4°C overnight. After washing three times to remove unbound peptides or antibodies, cells were incubated with 0.5 μg/ml PE-labeled streptavidin or goat anti-mouse IgG (Thermofisher, Waltham, MA) for 1 h at RT in the dark. After another three times washing, cell nuclei were stained by DAPI in mounting medium (1:500) (Invitrogen, Carlsbad, CA) and slides were viewed with and the images were recorded by a Zeiss LSM 5 LIVE line-scanning confocal microscope (Carl Zeiss, Oberkochen, Germany) at Harry S. Truman Veterans Memorial Hospital.

### Statistical analysis

Statistical comparisons were performed with Prism 6.0 GraphPad software (GraphPad Software Inc., San Diego, CA.). Results were compared with a two-sample Student's t test, two-way ANOVA multiple comparison test or chi-square test. All statistical tests were two-tailed tests and differences were considered significant at a *P* value of <0.05.
